# Efficiency of Pyroligneous Extract from *Jurema Preta* (*Mimosa tenuiflora* [Willd.] Poiret) as an Antiseptic in Cats (*Felis catus*) Subjected to Ovariosalpingohysterectomy

**DOI:** 10.3390/ani12182325

**Published:** 2022-09-07

**Authors:** Francisco Marlon Carneiro Feijo, Fernando da Costa Fernandes, Nilza Dutra Alves, Alexandre Santos Pimenta, Caio Sergio Santos, Gardenia Silvana de Oliveira Rodrigues, Alexsandra Fernandes Pereira, Leon Denner Moreira Benicio, Yasmin Beatriz França Moura

**Affiliations:** 1 Center of Agrarian Sciences, Federal Rural University of Semi-Arid Region—UFERSA, Mossoro 59625-900, Brazil; 2 Agricultural Sciences Academic Unit, Forest Sciences Graduate Program—PPGCFL, Forest, Bioenergy and Environment Research Group, Federal University of Rio Grande do Norte—UFRN, Natal 59078-970, Brazil; 3 Laboratory of Animal Biotechnology, Center for Biological and Health Sciences, Federal Rural University of Semi-Arid Region—UFERSA, Mossoro 59625-900, Brazil

**Keywords:** medicinal plants, antimicrobials, antiseptics, sterilized

## Abstract

**Simple Summary:**

The pyroligneous extract from *Mimosa tenuiflora* was used as an antiseptic in cats. The objective of this study was to analyze the action of 20% pyroligneous extract from *M. tenuiflora* as an antiseptic for surgical wounds of cats subjected to ovariohysterectomy. In addition to the absence of cytotoxicity, the number of bacteria was reduced after treatment. Tissue dehiscence, hyperemia, edema, crust, and secretions were not observed. Thus, the pyroligneous extract of *M. tenuiflora* has potential as a natural antiseptic alternative.

**Abstract:**

Pyroligneous extract of *Jurema preta* (*Mimosa tenuiflora* [Willd.] Poiret) was evaluated for its efficacy as a cutaneous antiseptic in cats (*Felis catus*) that were subjected to ovariosalpingohysterectomy. For this purpose, 30 cats without a defined breed were sterilized and divided into two groups. The first group was the positive control, treated with 0.5% chlorhexidine-alcohol solution, and the second group was treated with 20% pyroligneous extract of *M. tenuiflora*. Regardless of age and sex, all animals had visible healing at similar times. A significant reduction in bacterial growth was observed in animals treated with the extract, and no cytotoxicity was observed in the feline epithelial cells. In addition, surgical wounds of cats treated with *M. tenuiflora* extract exhibited improved healing. On agar plates, treatment with both chlorhexidine and *M. tenuiflora* extract resulted in the inhibition zones for all bacterial strains isolated from surgical wounds. Therefore, *M. tenuiflora* extract is demonstrated to have antiseptic effects on the surgical wounds of cats undergoing ovariosalpingohysterectomy.

## 1. Introduction

Brazil is in a privileged position in terms of biodiversity, comprising several biomes which includes Caatinga [1]. The Caatinga biome is in north-eastern Brazil, covering an extensive area, and is composed of a rich biodiversity of native plants with antimicrobial properties [2,3] due to their phytochemical components [4].

The use of plants for therapeutic purposes is one of the oldest forms of medicinal practices [5]. In the decade 2010–2020, there was an increase in the use of alternative therapeutic practices supported by policies, particularly the use of medicinal plants and herbal medicines. Ethnobotanical studies have mainly focused on plant species traditionally used for therapeutic applications [6]. Previously, research on the use of pyroligneous acid (PA) against *Escherichia coli*, *Staphylococcus aureus*, *Pseudomonas aeruginosa*, and yeasts *Candida albicans* and *Cryptococcus* neoformans was confirmed in vitro [7]. Another study analyzed the effect in vivo of PA derived from *Mimosa tenuiflora* as a cutaneous antiseptic in goats [8] and there were found a decrease in bacterial count in the wounds. Several other studies have been conducted for agricultural applications, organic insecticides, and fertilizers using PA [9,10,11].

PA is one of the most important liquid products obtained during wood pyrolysis. The pyrolysis products obtained from this process include PA, pyroligneous tar, pyrolysis oil, bio-oil, bio-crude oil, bio-fuel oil, liquid wood, wood oil, liquid smoke, and wood distillates [12].

Bioactive compounds such as phenol, guayacol, and furfural present in the pyroligneous acid extract act on the bacterial cell membrane, causing energy depletion and rupture of the membrane, thereby bringing about cell death [13] or the irreversible loss of membrane integrity as a phospholipid bilayer [14].

*M. tenuiflora* is a typical plant species in the semi-arid region of Brazil that has the potential to produce vegetable tannins [15]. Compounds such as catechol, caffeic acid, coumarin, rutin, quercetin, and kaempferol have been reported to be antibacterial agents in this plant [8].

The cutaneous antiseptic action of *M. tenuiflora* has been observed from extractions using the decoction method. The decrease in bacterial count in post-surgical wounds of cats after orchiectomy and ovariosalpingohysterectomy surgeries verified *M. tenuiflora* extract as an antiseptic [16].

Infectious wounds are common in surgeries such as ovariosalpingohysterectomy, and this is made more problematic in the presence of multidrug-resistant bacteria, such as *S. aureus*. Resistance to beta-lactams is common and is often due to the presence of genes such as blaZ and mecA [17]. Therefore, herbal medicines have emerged as potential alternative antiseptics. The present study aimed to evaluate the antiseptic efficacy of the pyroligneous extract of *M. tenuiflora* compared to conventional chemical antiseptics in surgical wound healing in cats (*Felis* *catus*) subjected to ovariosalpingohysterectomy.

## 2. Materials and Methods

### 2.1. Location of Experiment

The collected samples were processed at the Veterinary Microbiology Laboratory (LAMIV), whereas the surgical procedures for animal sterilization were performed in private clinics located in the municipality of Mossoro, Rio Grande do Norte, Brazil.

### 2.2. Production of Pyroligneous Extract

*M. tenuiflora* was obtained from the Center for Agrarian Sciences of the Federal University of Rio Grande do Norte, located in the city of Macaíba, Rio Grande do Norte. Wood wedges were dried in a greenhouse for 48 h at 103 ± 1 °C and 0% humidity. Shortly after, they were arranged in a metal container in batches of approximately 500 g, in which slow pyrolysis was performed in an electric kiln muffle equipped with a device designed to collect the condensable gases.

The condenser was cooled and maintained at 25 °C. Four carbonization sequences were performed at a heating rate of 1.25 °C per min until a final temperature of 450 °C was reached, which was maintained for 30 min. The total condensed liquid was kept at 2 °C until use. The four pyrolysis liquids formed the composite samples. This composite sample was bi-distilled via vacuum at 1.0 mmHg up to 100 °C, and the process was interrupted at temperatures above 102 °C. Wood tar and oil were discarded [12].

### 2.3. Action of Pyroligneous Extract In Vivo

From September 2019 to September 2020, 30 undefined breeds of *F. catus* were randomly selected. Their owners received information about the experiment and signed a consent form allowing surgery. The animals were subsequently anesthetized, subjected to ovariosalpingohysterectomy, and kept in the company of the tutors postoperatively until the healing of the surgical wounds.

The animals were divided into two randomized groups of 15 animals each. The first group was the positive control, which received 0.5% chlorhexidine-alcohol solution, and the second group received 20% extract solution of *M. tenuiflora*. The cutaneous lesion at the surgical site of each animal received either 1 mL of *M. tenuiflora* extract or 0.5% chlorhexidine-alcohol daily for seven days, and samples were collected daily from the surgical incision site with the aid of sterile swabs after 10 min of antiseptic action [16].

### 2.4. Bacterial Identification and Sensitivity Test

The bacteria isolated from the surgical site were cultured in BHI broth for 24 h at 37–37.5 °C until the log phase was reached for approximately 18–24 h and adjusted to the standard 0.5 McFarland scale. The bacterial identification was made according to MacFaddin (2000) [18] and the sensitivity test for the agar diffusion extracts was performed according to the National Committee for Clinical Laboratory Standards (2003) [19].

### 2.5. Bacteria Count

Cellular debris was collected via swabs and placed in a test tube containing 2 mL of sterile distilled water and subjected to dilutions of 1:10, 1:100, and 1:1000. After the procedure, 1 mL of each dilution was soaked in plate counting agar (PCA) and incubated in a bacteriological stove for 24 h at 37–37.5 °C for subsequent bacterial counts [20].

### 2.6. Clinical Aspects

The surgical wound site was observed in all animals for seven days in terms of the following clinical aspects: crusts, dehiscence, edema, hyperemia, and secretion. Data were annotated using a spreadsheet Excel [16].

### 2.7. Cytotoxicity

Fragments of the abdominal skin (9.0 mm^3^) obtained from a domestic cat submitted to ovariohysterectomy were cultured in vitro in Dulbecco’s Modified Eagle Medium supplemented with 10% fetal bovine serum and 2% penicillin-streptomycin solution at 38.5 °C and 5% CO_2_. The recovered cells were frozen in the second passage for toxicity experiments.

For toxicity analysis [8], cells were thawed and cultured in the third passage at 80% confluence and at a concentration of 5.0 × 10^4^ cells/mL. The cells were then divided into three experimental groups and incubated for 10 min according to each group: (a) without the presence of extract (PA0 group), (b) 20% *Jurema preta* extract (PA20 group) and (c) 2% iodine solution (group I2). After the incubation period, cell morphology was evaluated using an inverted microscope (Nikon TS100, Tokyo, Japan). In addition, cells were evaluated for viability using the 0.4% trypan blue assay; blue cells were considered non-viable and colorless cells were considered viable. Briefly, after trypsinization, the cells were incubated with trypan blue and counted in a Neubauer chamber, and the viability rate was calculated according to the following formula: (number of living cells/total number of cells counted) × 100. Subsequently, the metabolic activity of the cells was evaluated using 3-(4,5-dimethyl2-thiazolil)-2,5-diphenyl-2il-tetrazolic or MTT assays. The cells were then incubated with 5 mg/mL MTT for 3 h, after which the MTT solution was removed and 1 mL DMSO was added to solubilize the formazan crystals. Reading was performed using a spectrophotometer (Shimadzu^®^ UV-mini- 1240, Kyoto, Japan) at an absorbance of 595 nm.

### 2.8. Statistics

The microbiological analyses were submitted to variance analysis and the means were compared to each other by Tukey’s *t*-test at the level of 5% probability. Regarding the cytotoxicity data, expressed as ± standard error of four replicates, were analyzed using the GraphPad software 9.4.1 (GraphPad Software Inc., La Jolla, CA, USA). The identification of bacteria and clinical signs were tabulated in Excel tables. Regarding cytotoxicity, the results were verified for normality using the Shapiro–Wilk test and homoscedasticity using the Levene test. The data demonstrated no normal distribution and were transformed by using a sine arc. The results were analyzed by ANOVA (multiple comparisons) followed by Tukey’s test, at a significance level of *p* < 0.05.

## 3. Results and Discussion

**Figure 1:**
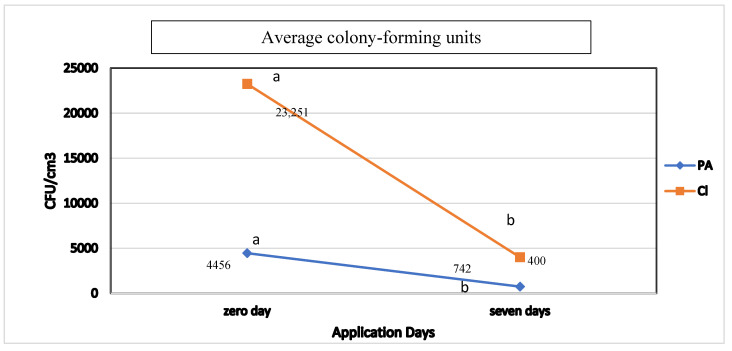
Average colony-forming units during bacterial count at Day 0 and 7 post-surgery when using 20% *M. tenuiflora* extract and 0.5% chlorhexidine in cats without defined breed subjected to sterilization surgery. CL = positive control (0.5% chlorhexidine-alcohol); PA = 20% Pyroligneous extract of *M. tenuiflora*. Means followed by different letters (a, b) represent significance (*p* < 0.05) in the treatment between days 0 and 7 according to Tukey’s test.

For animals treated with pyroligneous extract of *M. tenuiflora*, 4.5 × 10^3^ and 7.4 × 10^2^ bacterial cells were observed on day 0 and 7, respectively, with a reduction of 82%. In addition, those treated with chlorhexidine presented 2.3 × 10^4^ and 4.0 × 10^2^ bacterial cells on day 0 and 7, respectively, with a reduction of 83%. The data were significantly different (*p* < 0.05) when comparing *M. teinuflora* and chlorhexidine data. Both chlorhexidine and *M. tenuiflora* decreased bacterial cell counts (Figure 1).

The animals treated with *M. tenuiflora* demonstrated a significant reduction in bacterial growth, which is corroborated by Pimenta et al. (2018) [12], who reported that the pyroligneous extract of *M. tenuiflora* has a set of active ingredients with antimicrobial action. These active ingredients include phenolic compounds, such as 4-ethyl-2-methoxyphenol and 4-propyl-2-metoxifenols, that have antimicrobial properties [21] and most likely work by inactivating bacterial enzymes and destabilizing microbial cell membranes [22].

The results with 0.5% chlorhexidine also revealed inhibition of bacterial growth. Chlorhexidine is classified as a cationic detergent biguanide and comes in acetate, hydrochloride, and digluconate form. The digluconate form is the most commonly used salt in formulas and products and has a broad spectrum of action, acting on gram-positive and gram-negative bacteria, fungi, and yeasts [20].

The bacteria isolated from the surgical wounds of cats that underwent ovariosalpingohysterectomy are described in Table 1.

Among gram-negative bacteria, *Pantoea agglomerans*, a common bacterium in clinical diseases, is often isolated from skin lesions [23]. The second most prevalent was *Acinetobacter baumannii*, isolated from the skin of cats [24]. The action of *M. tenuiflora* on *A. baumanii* is particularly of interest, since this bacterium harbors virulence genes such as *afa/draBC*, *papC*, and *cvaC* [25] which are often neglected in veterinary medicine. The opportunistic pathogen *P. aeruginosa* was also isolated, which is expected since this bacterium is commonly present on the skin of humans and animals [26]. Another isolated pathogen was *Pseudomonas luteola* [27], which has been reported in pyogranulomatous panniculitis. The occurrence of *Pseudomonas spp.* in skin infections in cats is common because of its pathogenicity genes including *pelA*, *pslA*, *ppyR*, *fliC*, and *nan1* involved in the production of biofilms [28]. Herbal antimicrobials could act as an alternative to the traditional antibiotics currently used. The presence of *Moraxella spp.* was also verified and is justified by the fact that it is often isolated from the oral microbiota of dogs and cats (Sturgeon et al., 2014) [29]; therefore, its occurrence in the abdomen is probably due to the licking habits of feline species. Another study looked at chinese phytotherapeutic strains, which inhibited *Moraxella spp.* in vitro [30], specifically with regards to pyodermas of companion animals. Therefore, the pyroligneous extract of *M. tenuiflora* can be used as a possible antiseptic for surgical wounds, as suggested in the present study.

Gram-positive bacteria were also observed at the surgical sites, mainly *Staphylococcus* *lugdunensis* [31] and *Stomatococcus spp*., followed by *Staphylococcus* *saprophyticcus* and *Streptococcus spp*. These bacteria are highly resistant to opportunistic skin-domissanitary products [32] and are isolated from animals with otological problems [33]. The action of pyroligneous extract of *M. tenuiflora* against gram-positive bacteria has been observed in vitro [7] and in dairy animals [8]. The sensitivity of *S. aureus* is due to membrane lysis caused by the synergistic effect of the components of the pyroligneous extract of *M. tenuiflora* [34], which includes phenols, cresols, and 1,2-benzenodiol [35].

**Figure 2:**
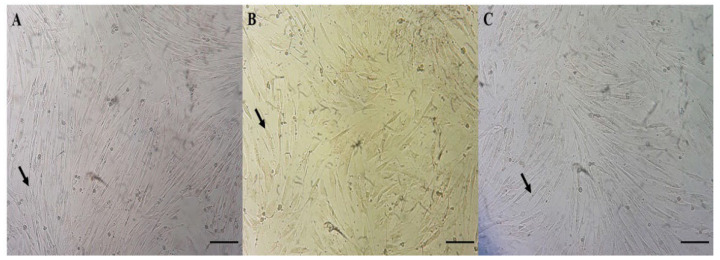
Morphological analysis of cells obtained from the abdominal region of domestic cat exposed to 20% *Jurema preta* extract and 2% iodine solution. (**A**) 0% *Jurema preta* (M. tenuiflora) extract (negative control), (**B**) 20% *Jurema preta* (*M. tenuiflora*) extract, and (**C**) 2% iodine solution at (positive control), 40×. Arrow indicates cells with fibroblast-like morphology.

After four replicate experiments, no difference was observed in the morphology of the incubated cells in the absence and presence of 20% *M. tenuiflora* extract and 2% iodine solution (Figure 2A–C). Monolayers of cells with fibroblast-like morphology and normal growth were observed in the treatment groups. The cells had spindle-shaped extensions and grew rapidly.

Regarding cell viability evaluated by the trypan blue assay (Table 2), 20% *Jurema preta* (*M. tenuiflora*) extract resulted in a higher percentage of viable cells compared to cells grown in the absence of extract and in 2% iodine solution (*p* < 0.05).

All groups showed an effect on metabolic activity. However, the 20% (*M. tenuiflora*) extract group was similar to that of the 2% iodine solution (Table 3). The viability of domestic cat epithelial cells grown in the presence of 20% (*M. tenuiflora*) extract was not altered, as evaluated by trypan blue staining. However, there was a reduction in metabolic activity in cells cultivated in the presence of 20% *M. tenuiflora* extract; thus, activity was similar to that observed in cells grown in 2% iodine solution. Studies examining PAs have demonstrated that these compounds have low toxicity (Campos, 2018) [30], which was also observed in the extract of *M. tenuiflora*. The pyroligneous extract of *M. tenuiflora* was similar to 2% iodine (*p* < 0.0001) in relation to metabolic activity and cell viability effects, suggesting its potential for use as an antiseptic in surgical wounds in cats. Moreover, Soares et al. [8] observed the potential of pyroligneous extract of *M. tenuiflora* as a goat antiseptic because it increased caprine cell viability.

**Figure 3:**
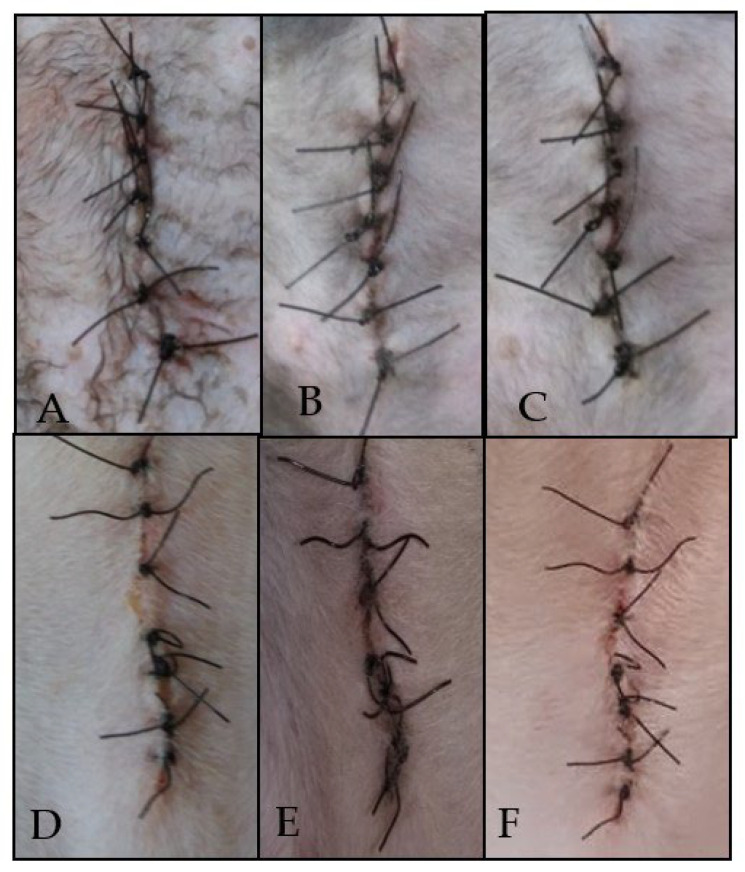
Action of pyroligneous extract *Mimosa tenuiflora* 20% on day 1 (**A**), day 3 (**B**), day 7 (**C**) and chlorhexidine to 0.5% on day 1 (**D**), day 3 (**E**) and day 7 (**F**) in surgical wounds of cats submitted to ovariosalpingohysterectomy.

In the present study, it was observed that the animals treated with 20% pyroligneous extract of *M. tenuiflora* and 0.5% chlorhexidine showed no symptoms of hyperemia, edema, secretion, crusts, or dehiscence. (Figure 3). A similar result was found by Soares et al. [8], who tested the pyroligneous extract of *M. tenuiflora* in the post-dipping of dairy goats. The authors reported satisfactory bacterial inhibition without alteration in the anatomophysiological structure of the teat or the physicochemical composition of the milk, most likely due to the capacity of the pyroligneous extract. These results are similar to those obtained using *Spondia mombim* at 100 mg/mL, and no alterations such as dehiscence, secretion, erythema, or edema were observed [16]. These effects are most likely inhibited by the anti-inflammatory action of phenolic compounds, such as ellagic and chlorogenic acids, present in plant extracts [36].

## 4. Conclusions

The 20% pyroligneous extract *Mimosa tenuiflora* is promising with natural antiseptic, inhibiting gram-positive and gram-negative bacteria, decreasing the bacteria count in surgical wounds of cats submitted to ovariosalpingohysterectomy. The product was not toxic to skin cells and it did not find hyperemia, edema, secretion, crusts, or dehiscence in surgical wounds.

## Figures and Tables

**Table 1:** Bacteria isolated from the surgical sites of cats subjected to ovariosalpingohysterectomy for antiseptic 20% PE of *M. tenuiflora* and 0.5% chlorhexidine.

Bacteria	Frequence	
	no.	%
Negative Gram	-	-
*Pantoea aglomerans*	5	26.31
*Acinetobacter baumannii*	3	15.78
*Pseudomonas aeruginosa*	2	10.52
*Pseudomonas luteola*	2	10.52
*Moraxela* spp.	1	5.26
Positive Gram	-	-
*Staphylococcus lugdunenses*	2	10.52
*stomatococcus* sp.	2	10.52
*Staphylococcus saprophyticcus*	1	5.26
*Streptococcus* spp.	1	5.26
*Aerococcus* sp.	1	5.26

**Table 2:** Cell viability evaluated by the trypan blue assay of cells from domestic cat grown in vitro after 10 min incubation with 20% *Jurema preta* extract (*M. tenuiflora*) and 2% iodine solution.

Groups	Cell Viability (%)	*p* in Relation to Control
Negative Control (PA0)	64.2 ± 3.3 ^b^	-
PA 20% (PA20)	76.1 ± 5.3 ^a^	0.028
2% Iodine Solution (I2)	56.8 ± 3.3 ^b^	0.188

Values expressed as average ± standard deviation (SD). a, b: differ (*p* < 0.05).

**Table 3:** Metabolic activity evaluated by MTT assay of domestic cat cells grown in vitro after 10 min of incubation with 20% *Jurema preta* (*M tenuiflora*) extract or 2% iodine.

Groups	Metabolic Activity (%)	*p* in Relation to Control
Negative Control (PA0)	100.0 ± 1.9 ^a^	-
20% *M. tenuiflora* extract (PA20)	49.5 ± 1.3 ^b^	0.0001
2% Iodine Solution (I2)	57.4 ± 2.7 ^b^	0.0001

Values expressed as average ± standard deviation (SD). a, b: significant difference (*p* < 0.05).

## Data Availability

The data presented in this study are available upon reasonable request from the corresponding author.
